# Baculovirus IE2 Stimulates the Expression of Heat Shock Proteins in Insect and Mammalian Cells to Facilitate Its Proper Functioning

**DOI:** 10.1371/journal.pone.0148578

**Published:** 2016-02-10

**Authors:** Hsuan Tung, Sung-Chan Wei, Huei-Ru Lo, Yu-Chan Chao

**Affiliations:** 1 Molecular and Biological Agricultural Sciences, Taiwan International Graduate Program, Academia Sinica, Taipei, Taiwan, and National Chung Hsing University, Taichung, Taiwan; 2 Institute of Molecular Biology, Academia Sinica, Taipei, Taiwan; 3 Graduate Institute of Biotechnology, National Chung Hsing University, Taichung, Taiwan; 4 Graduate Institute of Life Sciences, National Defense Medical Center, Taipei, Taiwan; 5 Biotechnology Center, National Chung Hsing University, Taichung, Taiwan; Wuhan Bioengineering Institute, CHINA

## Abstract

Baculoviruses have gained popularity as pest control agents and for protein production in insect systems. These viruses are also becoming popular for gene expression, tissue engineering and gene therapy in mammalian systems. Baculovirus infection triggers a heat shock response, and this response is crucial for its successful infection of host insect cells. However, the viral protein(s) or factor(s) that trigger this response are not yet clear. Previously, we revealed that IE2-an early gene product of the baculovirus-could form unique nuclear bodies for the strong *trans*-activation of various promoters in mammalian cells. Here, we purified IE2 nuclear bodies from Vero E6 cells and investigated the associated proteins by using mass spectrometry. Heat shock proteins (HSPs) were found to be one of the major IE2-associated proteins. Our experiments show that HSPs are greatly induced by IE2 and are crucial for the *trans*-activation function of IE2. Interestingly, blocking both heat shock protein expression and the proteasome pathway preserved the IE2 protein and its nuclear body structure, and revived its function. These observations reveal that HSPs do not function directly to assist the formation of the nuclear body structure, but may rather protect IE2 from proteasome degradation. Aside from functional studies in mammalian cells, we also show that HSPs were stimulated and required to determine IE2 protein levels, in insect cells infected with baculovirus. Upon inhibiting the expression of heat shock proteins, baculovirus IE2 was substantially suppressed, resulting in a significantly suppressed viral titer. Thus, we demonstrate a unique feature in that IE2 can function in both insect and non-host mammalian cells to stimulate HSPs, which may be associated with IE2 stabilization and lead to the protection of the its strong gene activation function in mammalian cells. On the other hand, during viral infection in insect cells, IE2 could also strongly stimulate HSPs and ultimately affect viral replication.

## Introduction

Baculoviruses are popular microbial control agents used to regulate insect pest populations in the field [1]. Since 1983, baculoviruses have become established as a widely-used eukaryotic protein production system-known as the baculovirus expression vector system-for laboratory and industrial applications [2]. Other than for gene expression in insect cells, baculoviruses can also serve as efficient gene delivery and protein expression tools in mammalian cells with the assistance of recognizable mammalian promoters [3]. Baculoviruses provide advantages over various other gene delivery methods in mammalian systems. Due to their stringent host range that only allows baculoviruses to replicate in insect cells and not in mammalian cells, all baculoviral genes are either not expressed or expressed at very low levels in mammalian cells [4, 5]. Thus, in contrast to mammalian vectors, baculoviruses incur much less biosafety and pathogenic concerns for human applications. In addition, cytotoxicity is low even with the use of a high multiplicity of infection (MOI). Baculoviruses have a large capacity for recombinant inserts, and multiple expression cassettes can be introduced together into a single virus or introduced separately into multiple viruses then infect cells jointly to result multiple protein expression, this greatly enabling complex protein production [6]. The applications of baculoviruses have been widely expanded to tissue engineering [7], gene therapy [8], and the formation of virus-like particles [9] for vaccine production [10, 11].

Among hundreds of baculoviruses, *Autographa californica* multicapsid nucleopolyhedrovirus (AcMNPV) is an extensively-studied species and has a long history as a highly-versatile vector for insect cell protein production. AcMNPV is a DNA virus with a circular genome of 134 kb, containing 155 open reading frames [12]. During its life-cycle in infected insect cells, gene expression proceeds in a rhythmic fashion that can be divided into four temporally-ordered phases: immediate-early, delayed-early, late and very late. The immediate-early genes do not require viral factors for expression, and they are believed to start the transcriptional cascade that initiates the baculovirus infection cycle as they are responsible for the activation of subsequent genes. Delayed-early genes are dramatically activated by immediate-early gene products, such as IE1, and are mostly involved in virus replication. The late and very late genes are transcribed by virally-encoded RNA polymerases and are usually expressed at a high level [13].

Baculovirus IE2 is one of the immediate early genes that are expressed right after baculovirus infection. Since IE2 is expressed even earlier than IE1 [14], it is regarded as an important factor in the regulation of baculovirus infection. As a transcriptional activator, IE2 activates a number of baculovirus genes during the virus life-cycle, including itself, *ie1*, *39k*, *etl*, *vp39* and *polh* [15–17]. IE2 protein interacts with itself through its C-terminal coiled-coil domain [18], and transiently forms nuclear bodies in the early phase of the infection cycle. The formation process is highly regulated by the IE2 oligomerization and ubiquitin ligase functional domains [14]. IE2 has a stimulating effect on virus replication [19], and the nuclear bodies have been found to be related to the site of virus replication where IE2 co-localizes with several other viral factors, such as DBP and LEF3 [20].

We have previously shown that when properly expressed by a mammalian promoter, IE2 still possess its activator function in mammalian cells [4]. We have also found that it is capable of strongly boosting mammalian promoters, such as the expression of CMV immediate early (IE) and SV40 promoters in both Vero E6 and U2OS cells [4]. This activation can be further augmented by the presence of the baculovirus enhancer element, the *hr* sequence [4]. Unlike conventional transcriptional factors, it is doubtful that IE2 achieves activation via direct binding to the promoter. In an extensive analysis of *Orgyia pseudotsugata* MNPV IE2, a specific sequence required for IE2 *trans*-activation could not be identified [21], suggesting that IE2 does not bind to a specific sequence to achieve activation. Instead, we have found that IE2 achieves strong activation by forming a novel nuclear body structure to recruit active RNA polymerase II and nuclear actin, producing a high concentration of mostly viral RNA inside the structure [4]. Therefore, the IE2 nuclear body has been regarded as an active transcription center for locally concentrating transcription materials, leading to strong activation. Given that the application of baculoviruses is usually restricted to a limited expression level in mammalian systems [22], these discoveries can greatly advance the application of baculoviruses as a valid tool for gene expression in these heterologous systems.

To further investigate the regulatory mechanism of IE2 *trans*-activation, we purified IE2 nuclear bodies and identified IE2-associated protein using mass spectroscopy (MS). We found that heat shock proteins (HSPs) are one of the major components that complex with the IE2 protein. Stimulation of a heat shock response during baculovirus infection and its importance for virus amplification has been frequently mentioned in the literature [23–26]. However, the viral factors that are responsible for this effect and the mechanism underlying the interaction between viral factors and HSPs are still unclear [27]. Here, we show that by inhibiting the heat shock response, IE2 protein became degraded and could not accumulate. This led to the failure of IE2 nuclear body formation and the loss of its *trans*-activation activity in mammalian cells. Upon blocking the proteasome-dependent degradation pathway, IE2 nuclear bodies could form and their proper functioning was restored. These findings were further confirmed in the original baculovirus host insect cells, where we found that IE2 was not only the stimulator of HSPs expression, but that the interaction between IE2 and heat shock proteins also ensured proper virus propagation. Thus, we have identified a crucial function of IE2, demonstrated a delicate interaction between heat shock proteins and the ubiquitin-proteasome pathway during baculovirus infection, and provided insight into the long-standing question regarding the role and importance of heat shock responses during baculovirus infection.

## Materials and Methods

### Cells, virus, generation of anti-IE2 antiserum

Vero E6 cells were cultured in MEM-alpha medium containing 10% FBS at 37°C with 5% CO_2_. *Spodoptera frugiperda* IPLB-Sf21 (Sf21) cells were grown at 26°C in TC100 insect medium containing 10% FBS. Recombinant AcMNPV was generated and propagated in Sf21 cells according to standard protocol [28]. The virus titers were determined by quantitative PCR [29]. Anti-IE2 serum was generated against synthetic peptide NSENVDRERFPDITC, followed by immunization into rabbits (GenesScripts).

### Plasmid and virus construction

The primers used in plasmid and virus construction are provided in S1 Table. Recombinant baculoviruses vAcIE2, vAcIE2C230S and vAcE-which express wild-type IE2, RING domain mutant IE2 and EGFP, respectively-were generated as previously described [4]. The *Luciferase* gene was obtained by PCR from pGL-3 (Promega) using primer Luc-NcoI-F and Luc-SacI-R, before being inserted into pTriEx-3 to generate pAcL.

Construction of pKShE was as described previously [30]. To generate IE2-expressing plasmid for the insect system, pKShIE2, the AcMNPV *ie2* gene was amplified from pAcIE2 using IE2-F and IE2-R primers and inserted into linearized vector, which was amplified from pKShE by primers pKShE-F and pKShE-R, excluding the *egfp* gene. For the IE1 dynamic study in Sf21 cells, IE1 CDS and its promoter were amplified from total AcMNPV genomic DNA using primesr pIE1-F and IE1-R, before being inserted into pBacPAK8 (Clontech), linearized by PCR amplification using primers pBacPAK-F and pBacPAK-R, resulting in pABiIE1. The *Wasabi* gene was amplified from pmWasabi-Actin (Alele Biotechnology) using primers L2-W-F and W-FLAG-R to attach an L2 linker at its N-terminal and a Flag tag at its C-terminal ends. The tagged *wasabi* gene was then inserted into pABiIE1, linearized by PCR amplification using primers pABiIE1-F and pABiIE1-R, resulting in pABiIE1WF. The In-Fusion HD Cloning kit (Clontech) was used to generate the aforementioned constructs according to the manufacturer’s manual.

Recombinant viruses were produced by co-transfecting pAcL or pABiIE1WF with vAcRP23.Laz (Pharmingen)-a linearized viral DNA of AcMNPV-into Sf21 by Cellfectin (Life Technologies), resulting in vAcL and vABiIE1WF, respectively. In these recombinant baculoviruses, the IE2, RING domain mutant IE2, EGFP and luciferase gene products expressed by vAcIE2, vAcIE2C230S, vAcE and vAcL, respectively, were driven by the TriEX promoter. This is a composite promoter containing both the *p10* and the CMV promoters, which are capable of driving gene expression in insect and mammalian cells, respectively [4]. The IE1 gene product expressed by the recombinant baculovirus vABiIE1WF was driven by the *ie1* promoter.

### Transduction and transfection of mammalian cells

Vero E6 cells were seeded overnight prior to transduction, and recombinant baculovirus was added directly into the culture medium at an MOI of 50, followed by centrifugation at 2,000 rpm for 32 min and incubation at 37°C with 5% CO_2_ for 48 h before being collected for further experiments.

For small interfering RNA (siRNA) experiments, HSP 70 siRNA, HSP 90α/β siRNA and control siRNA were purchased from Santa Cruz Inc. Transfection was performed using TransIT-siQUEST following the manufacturer’s instructions (Mirus). After an overnight incubation, fresh medium containing recombinant baculovirus was used to replace the siRNA-containing medium. The cells were incubated for an additional 48 h before the luciferase assay or Western blotting assay.

### Infection and transfection of insect cells

Sf21 cells were seeded for 1 h before vAcE was added into culture medium at MOI of 20, followed by centrifugation at 2,000 rpm for 30 min. For plasmid transfection, DNA was introduced into cells by Cellfectin (Life Technologies) according to the manufacturer’s protocol.

### IE2 nuclear body purification and Mass spectrometry

vAcIE2-transduced Vero E6 cells were collected at 48 hours post-transduction (hpt). Then they were washed twice with DPBS buffer and lysed in EMBK/0.1% NP-40 buffer (25 mM HEPES, pH 7.6, 5 mM MgCl_2_, 1.5 mM KCl, 75 mM NaCl, 175 mM sucrose, 0.1% NP-40, and protease inhibitors) at 4°C for 30 min [31]. The nuclei were collected by centrifugation at 1,400 ×g for 5 min and washed twice with EMBK without NP-40. The nuclei were suspended in 0.35 M sucrose and sonicated with a Misonix 2020 sonicator. DNaseI (1:100) and heparin (4 mg/ml) were added to the nuclear extract before incubation at room temperature overnight. The nuclear extract was then added to a 45% CsCl solution followed by ultracentrifugation at 43,000 rpm with a Bechman coulter SW60 Ti rotor for 48 h. Each fraction was spotted onto a poly-L-lysine-coated glass microscope slide to detect the presence of the IE2 nuclear bodies. The slides were air-dried, rehydrated in PBS, and labeled with various antibodies. The IE2-containing fraction was then dialyzed overnight into 50 mM Tris buffer and concentrated by centrifugation.

To identify the IE2-associated proteins, 1/10 volume of 0.1M DTT was first added to the protein solution, and the reaction was incubated at 37°C for 30 min. 1/10 volume of 0.55M IAA was then added, followed by incubation in the dark at 30°C for 30 min. Modified sequencing-grade trypsin (ratio of 50:1) was applied to the solution with the substrate, followed by incubation overnight at 37°C. To stop the reaction, the pH was adjusted to below 6, and then salts were removed by a Millipore Zip-tip.

LTQ-Orbitrap XL was used to analyze the tryptic peptides. The MS and MS/MS raw data were processed by Raw2MSM v. 1.10 and searched against an in-house-generated Primate-AcMNPV database using a Mascot Daemon 2.5.1 server. The search criteria were trypsin digestion, with variable modifications set as carbamidomethyl (C) and oxidation (M) and allowing up to 2 missed cleavages, a mass accuracy of 10 ppm for the parent ion and 0.60 Da for the fragment ions. A false discovery rate (FDR) of 1% was adopted.

### Immunoprecipitation and Immunoblotting

The nuclear extracts were first incubated with anti-His antibodies (1:200, Abcam ab18184) and protease inhibitor (Calbiochem) overnight at 4°C with continuous mixing on a rotating platform, followed by the addition of protein G magnetic beads (Millipore) for another 1 h at room temperature. The beads were washed in DPBS with 0.1% Tween 20 and then the proteins were eluted with Laemmli buffer and heated in boiling water for 10 min before electrophoresis. For Western blotting, anti-IE2 serum (1:3000), anti-HSP70 (1:3000, Genetex GTX111088), anti-Ubiquitin (1:1000, Santa Cruz sc-8017), anti-actin antibody (1:3000, Genetex GTX109639), or anti-GAPDH (1:3000, Genetex GTX100118) were used for the detection of target protein.

### RNA extraction of RT-qPCR

Total RNA was extracted using an RNeasy Mini kit (Qiagen) and reverse-transcribed using SuperScript III (Life Technologies). The quantity and quality of RNA was checked using a NanoDrop ND-1000 spectrophotometer (Thermo Fisher). The amounts of indicated transcripts were measured by quantitative PCR using specific primers (see S1 Table).

### Drug assay

KNK437 was purchased from Calbiochem, and Prostaglandin A_1_ (PGA_1_) and MG132 were procured from Sigma. For Vero E6 cells, cells transduced with the indicated recombinant baculovirus were treated with various drugs at 0 hpt at indicated concentrations. For Sf21 cells, cells were first incubated with 200 μM KNK437 for 24 h before adding virus into the medium. Cells were incubated in the presence of drugs until they were fixed or harvested at indicated time-points.

### Luciferase assay

A detailed protocol for the luciferase assay has previously been described [4, 32, 33]. Briefly, the cells were washed with Dulbecco’s phosphate-buffered saline (DPBS; Life Technologies) and lysed with 100 μl of cell culture lysis reagent (CCLR). The cell lysates were centrifuged at 3,500 rpm for 30 min at 4°C. Twenty microliters of the supernatant with 180 μl of luciferase assay reagent were placed into the wells of a black 96-well microplate. Luciferase activity was measured with a luminometer (EnSpire^®^, PerkinElmer) by injecting 50 μl of 0.2 mM luciferin (Promega) into each well.

### Immunofluorescence staining

Vero E6 or Sf21 cells were seeded onto an 8-well Millicell^**®**^ EZ slide (4 ×10^4^/well), and the cells were transduced with recombinant baculovirus. The cells were fixed and stained as described previously [4]. Briefly, the cells were fixed with 4% paraformaldehyde, and then permeabilized with 100% acetone at -20°C. After blocking with 3% BSA for 1 h, the cells were then incubated with primary antibody overnight at 4°C. Primary antibodies anti-IE2 antiserum (1:10000), anti-His antibodies (1:10000, Abcam ab18184), anti-FLAG (1:500, Sigma F1804), anti-HSP70 (1:5000, Genetex GTX111088) or anti-HSP90 alpha (1:2000, Genetex GTX109753) were diluted in blocking buffer. After incubation overnight, the cells were washed three times with DPBST (DPBS containing 0.1% Tween 20) and then incubated for 1 h with secondary antibodies including Dylight goat 549 anti-rabbit IgG, Dylight 405 goat ani-mouse IgG (1:200, Jackson) or Alexa Fluor 488 goat anti-mouse IgG (1:500; Life Technologies). Also, Alexa Fluor 488-conjugated DNaseI (1:500; Life Technologies) was used for G-actin detection, and Hoechst dye (Life Technologies) for nucleus staining. After washing three times with DPBST, cells were sealed with mounting solution (Fluoromount-G, SouthernBiotech). Images were collected using a Zeiss laser confocal microscope (LSM 780).

## Results

### HSP70 and HSP 90 associate with IE2 in Vero E6 cells

**Fig 1:**
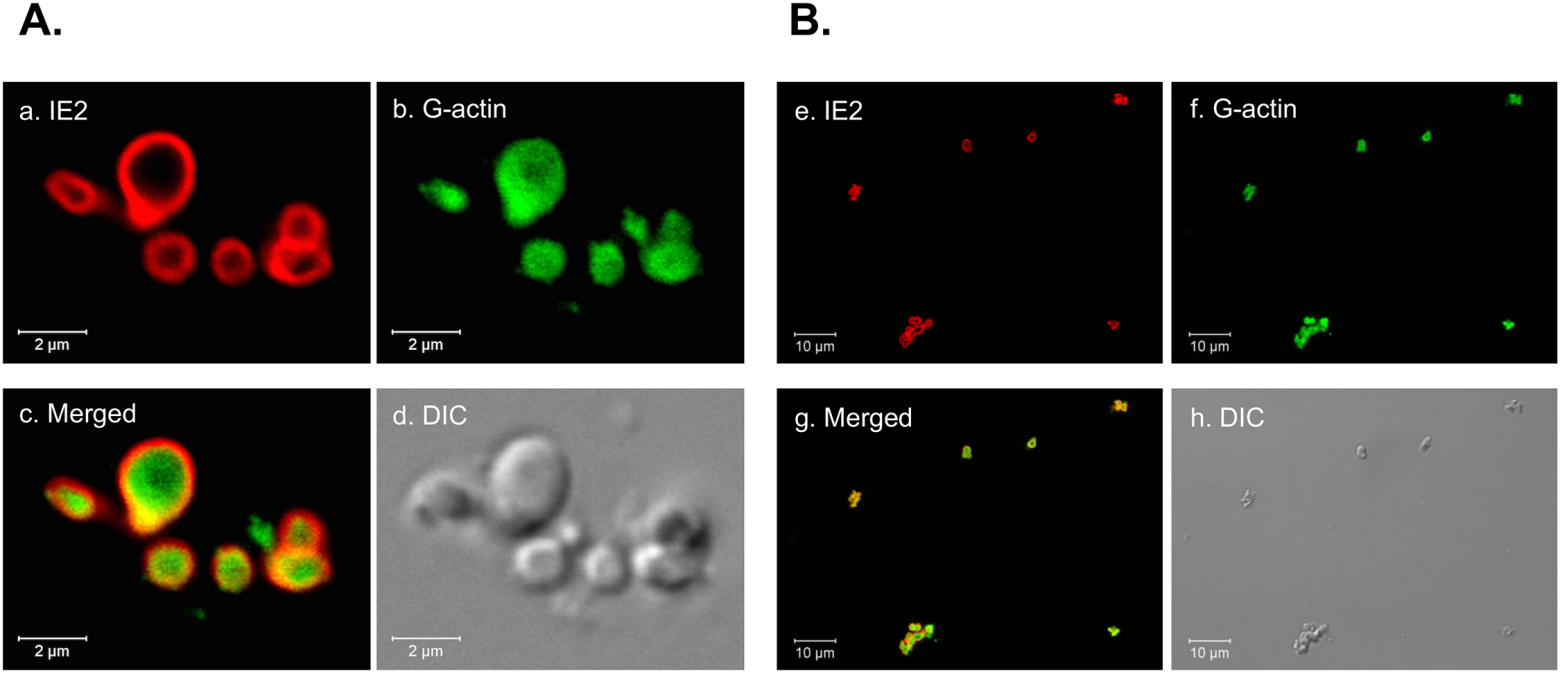
Purification of the IE2 nuclear bodies. Fractions containing IE2 protein were spotted onto poly-L-lysine-coated glass slides and immobilized with antibody and DNaseI for the detection of IE2 and G-actin, respectively. Panels *a* to *d* show that numerous IE2 nuclear bodies maintained their integrity after the purification procedure. Panels *e* to *h* show that the fraction largely contains IE2 nuclear bodies with little other visible cell debris.

To identify the proteins that interact with IE2, Vero E6 cells were transduced with the IE2-expressing virus, vAcIE2, for 48 h. The transduced cells were then collected and the nuclear extract was subjected to cesium chloride (CsCl_2_) density gradient centrifugation to isolate IE2 nuclear body particles. The fractions were analyzed for the presence of IE2, as well as for the integrity of the nuclear body structure, by immunofluorescence staining. As shown in Fig 1, the IE2 nuclear body structure and the protein components inside the structure (where we used G-actin as an indicator of structural integrity [4]), remained intact under the purification procedure that we employed. LTQ-Orbitrap XL (Thermo) was then used to analyze the tryptic peptides generated following purification. Among the highest-ranking proteins that were specifically present in the IE2 fraction from each of several independent experiments were HSPs. Table 1 shows a representative result from one of the experiments. Several members of the HSP70 family were detected with significantly higher scores, while the HSP90 family members were also clearly detected, although with relatively lower scores.

**Table 1:** Summary of selected MS-identified proteins from the IE2 nuclear body complex.

Accession number	Protein	Species	Mol. mass	Protein sequence coverage	No. of significant peptides	Moscot Score
P24647	E3 ubiquitin-protein ligase IE2	*Autographa californica* nuclear polyhedrosis virus	46978	47.5%	14	385
Q4R561	Actin	*Macaca fascicularis*	41710	54.7%	15	295
P11142	Heat shock cognate 71 kDa protein	*Homo sapiens*	70854	23.1%	5	181
P54652	Heat shock-related 70 kD protein 2	*Homo sapiens*	69978	17.1	4	149
P08107	Heat shock 70 kDa protein 1A/1B	*Homo sapiens*	70009	10.6	4	132
Q5NVM5	60 kDa heat shock protein	*Pongo abelii*	60959	26	6	129
P34931	Heat shock protein 70kD 1-like	*Homo sapiens*	70331	7.5	3	125
P17006	Heat shock protein 70kD 6	*Homo sapiens*	70984	10.6	3	119
Q28222	Heat shock protein 70kD 1	*Chlorocebus aethiops*	69877	8.2	3	93
P48741	Putative heat shock 70 kDa protein 7	*Homo sapiens*	40220	11.7	2	81
P04792	Heat shock protein beta-1	*Homo sapiens*	22768	4.9	1	63
Q5R710	Heat shock protein HSP 90-beta	*Pongo abelii*	83186	7.3	1	41

**Fig 2:**
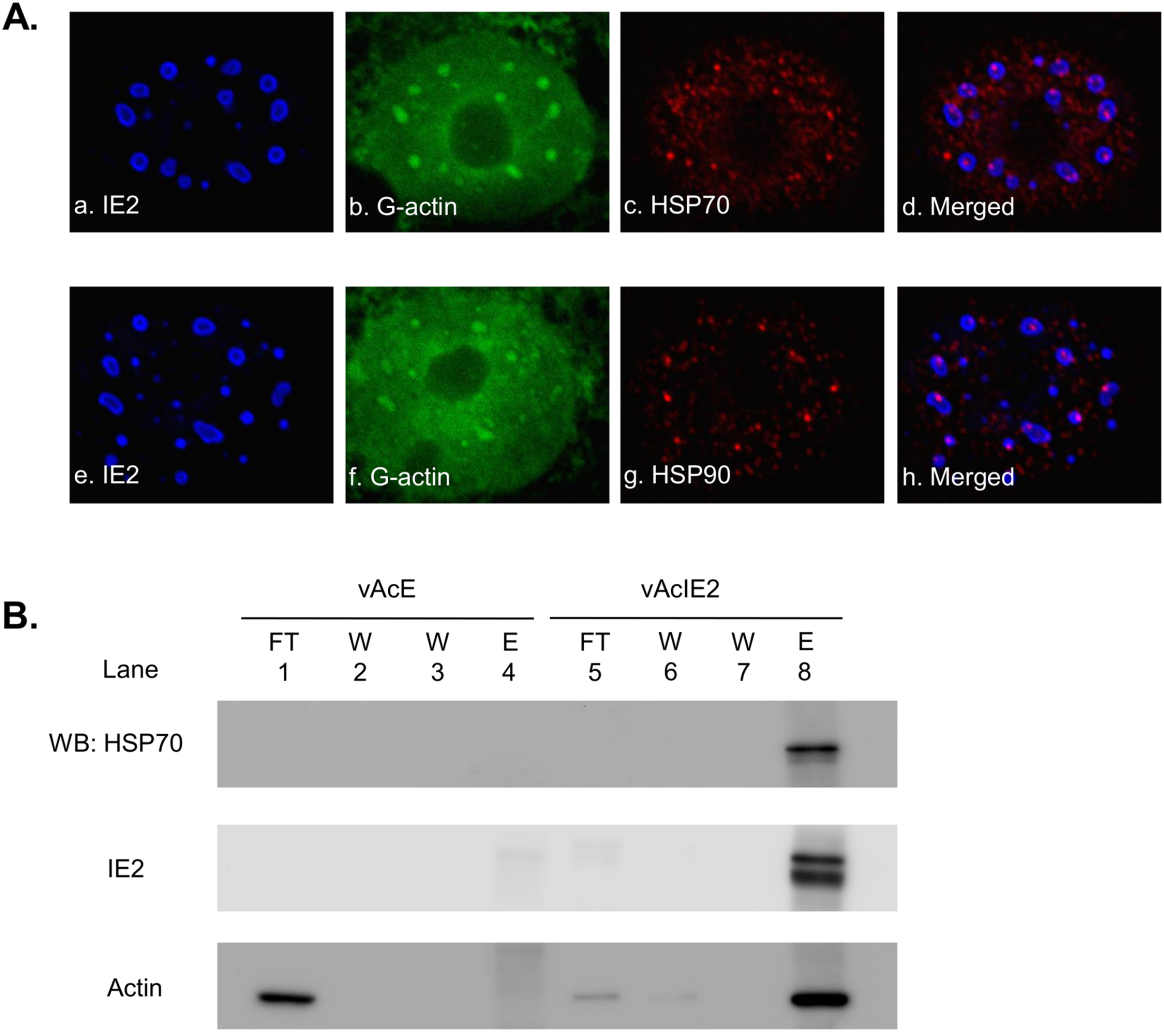
IE2 associates with HSPs. vAcIE2-transduced Vero E6 cells were fixed and examined by immunofluorescence staining at 48 hpt. Nuclei have been enlarged to more clearly present the interaction between IE2, HSP70 and HSP90. (A) Panels *a* to *d* show that HSP70 formed dots and was scattered within the nucleus. Larger foci of HSP70 closely associated with IE2 nuclear bodies. Panels *e* to *h* show that HSP90 also formed foci and was closely associated with IE2 nuclear bodies. (B) The IE2 protein complex was immunoprecipitated using an anti-histidine antibody from nuclear extracts of the vAcIE2-transduced Vero E6 cells. Nuclear extracts of the vAcE-transduced Vero E6 cells were used as a control. HSP70, actin and IE2 were detected by Western blotting using the corresponding antibody. FT: flow-through, W: wash, E: eluted fraction.

To confirm the interaction between HSPs and IE2, their association was examined by immunoprecipitation and Western blotting. The cellular distribution of HSP70 and HSP90 were also examined by immunofluorescence. As shown in Fig 2A, HSP70 and HSP90 formed foci that closely associated with the IE2 nuclear bodies within the nucleus. Fig 2B shows that nuclear actin was largely concentrated in the IE2 fraction, which is consistent with our previous fractionation results showing that IE2 associates with a high concentration of nuclear actin [4]. Corresponding to the results from the mass spectrometry analysis, HSP70 also co-precipitated with IE2 in the elution fraction, confirming a direct interaction between HSP70 and IE2. However, we were unable to detect an HSP90 signal using the immunoprecipitation method, which indicates a relatively weak or transient interaction between HSP90 and its client protein [34].

### IE2-expressing recombinant virus transduction stimulates HSP70 and HSP90 expression in Vero E6 cells

**Fig 3:**
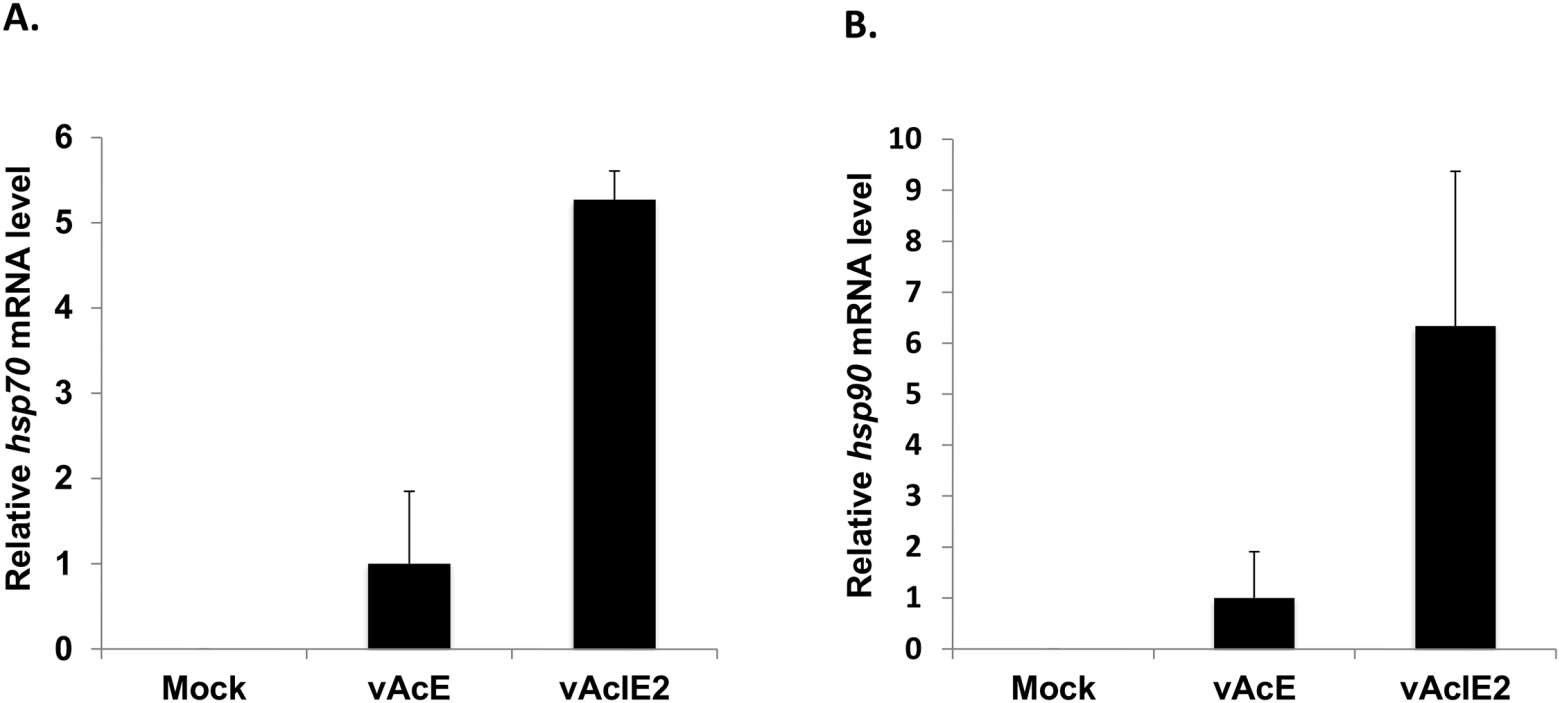
IE2 stimulates HSP70 and HSP90 expression in Vero E6 cells. Total RNA was extracted from vAcIE2-transduced Vero E6 cells at 6 hpt, and the transcripts of *hsp70* (A) and *hsp90* (B) were quantified by real-time quantitative PCR. The expression levels of *hsp70* and *hsp90* have been normalized to that of *18s* rRNA. Total RNA from vAcE-transduced and non-transduced cells served as the controls.

The heat shock response is frequently stimulated by stresses to maintain cellular homeostasis [35]. One of the most obvious indicators of the heat shock response is the up-regulation of HSP expression. We investigated whether the transduction of an IE2-expressing recombinant virus, where IE2 is driven by the CMV promoter, can stimulate HSP expression in Vero E6 cells. The mRNA levels of the two most important members of the HSP family (HSP70 and HSP90) were quantified using RT-qPCR. As shown in Fig 3, although the control virus expressing a foreign protein (EGFP, which is also driven by the CMV promoter), resulted in slightly increased *hsp70* and *hsp90* expression, the IE2-expressing virus led to five-fold and six-fold increases in *hsp70* and *hsp90* transcript expression compared to the control virus, respectively. These results indicate that IE2 is a strong stimulator of HSP expression during baculovirus transduction.

### Heat shock proteins positively regulate IE2 *trans*-activation activity in Vero E6 cells

**Fig 4:**
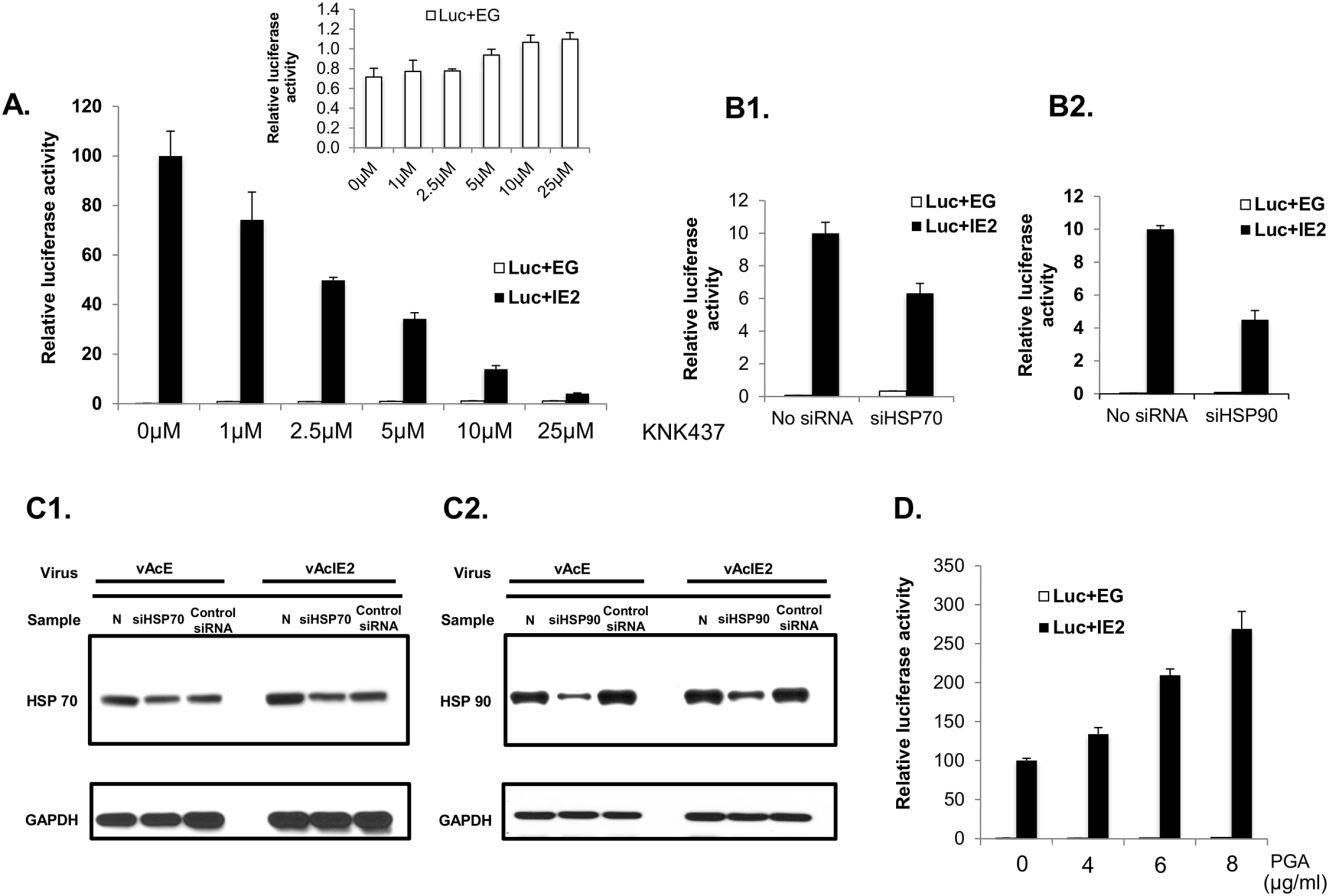
HSPs positively regulate IE2 transaction activity. (A) vAcIE2 and vAcL co-transduced Vero E6 cells were treated with KNK437 at various concentrations, followed by luciferase assays at 48 hpt. The results show that IE2 *trans*-activation activity on the CMV promoter was down-regulated in a dose-dependent manner. KNK437 did not have a negative effect on the control group represented by vAcE transduction. (B) siRNA against *hsp70* (B1) and *hsp90* (B2) genes were transfected into Vero E6 cells, followed by vAcIE2 and vAcL co-transduction. A luciferase assay at 48 hpt detected negative effects on the IE2 *trans*-activation of the CMV promoter. (C) siRNA against *hsp70* (C1), *hsp90* (C2) and negative control siRNA against a scrambled sequence (Santa Cruz) were transfected into Vero E6 cells, followed by vAcIE2 or vAcE transduction. Cell extracts were harvested at 48 hpt and analyzed by Western blotting. (D) PGA_1_, which stimulates the heat shock response, further enhanced the IE2 *trans*-activation effect on the CMV promoter in a dose-dependent manner.

To understand the biological function of HSPs for IE2 *trans*-activation activity, the heat shock response was either blocked or alleviated using various methods. First, a benzylidene lactam compound, KNK437-the most widely-used heat shock response inhibitor [23, 36–38]-was applied to our experiments. KNK437 prevents the development of thermotolerance by inhibiting the up-regulation of various HSPs at the transcriptional level [39]. In response to KNK437 treatment, IE2 activation levels decreased significantly in a dose-dependent manner (Fig 4A), whereas treatment did not result in a decrease in general transgene expression, as shown in the control group represented by vAcE-transduced cells. The slight up-regulation observed for the control group might have arisen from the apoptotic effect of KNK437 [38], leading to stimulation of the CMV promoter used in our system [40]. To confirm the observed effect, two of the major proteins in the heat shock response-HSP70 and HSP90-were also specifically knocked down using siRNA. Fig 4B shows that upon siRNA knock-down, IE2 *trans*-activation activity decreased. In order to analyze the levels of HSPs upon siRNA treatment, siRNAs against *hsp70* (Fig 4C1) and *hsp90* (Fig 4C2) were transfected into Vero E6 cells, followed by vAcE or vAcIE2 co-transduction at 24 h post siRNA transfection. Levels of protein expression were then examined by Western blotting. Each siRNA had a different effect on the level of knock-down for the respective protein. siHSP70 down-regulated HSP70 expression by about 20% (Fig 4C1), whereas siHSP90 knocked down HSP90 expression much more significantly, by about 40% (Fig 4C2). These data echo the data shown in Fig 4B where siHSP90 treatments were more effective in down-regulating IE2 *trans*-activation activity than siHSP70.

We confirmed this effect by inducing the heat shock response using PGA_1_, which is known to induce overexpression of HSP70 by increasing the DNA binding affinity of the heat shock factor [41]. The result showed that IE2 *trans*-activation was enhanced upon PGA_1_ treatment in a dose-dependent manner (Fig 4D). Together, our results indicate a positive role for HSPs in IE2 *trans*-activation activity.

### Heat shock proteins play crucial roles in IE2 protein stability

**Fig 5:**
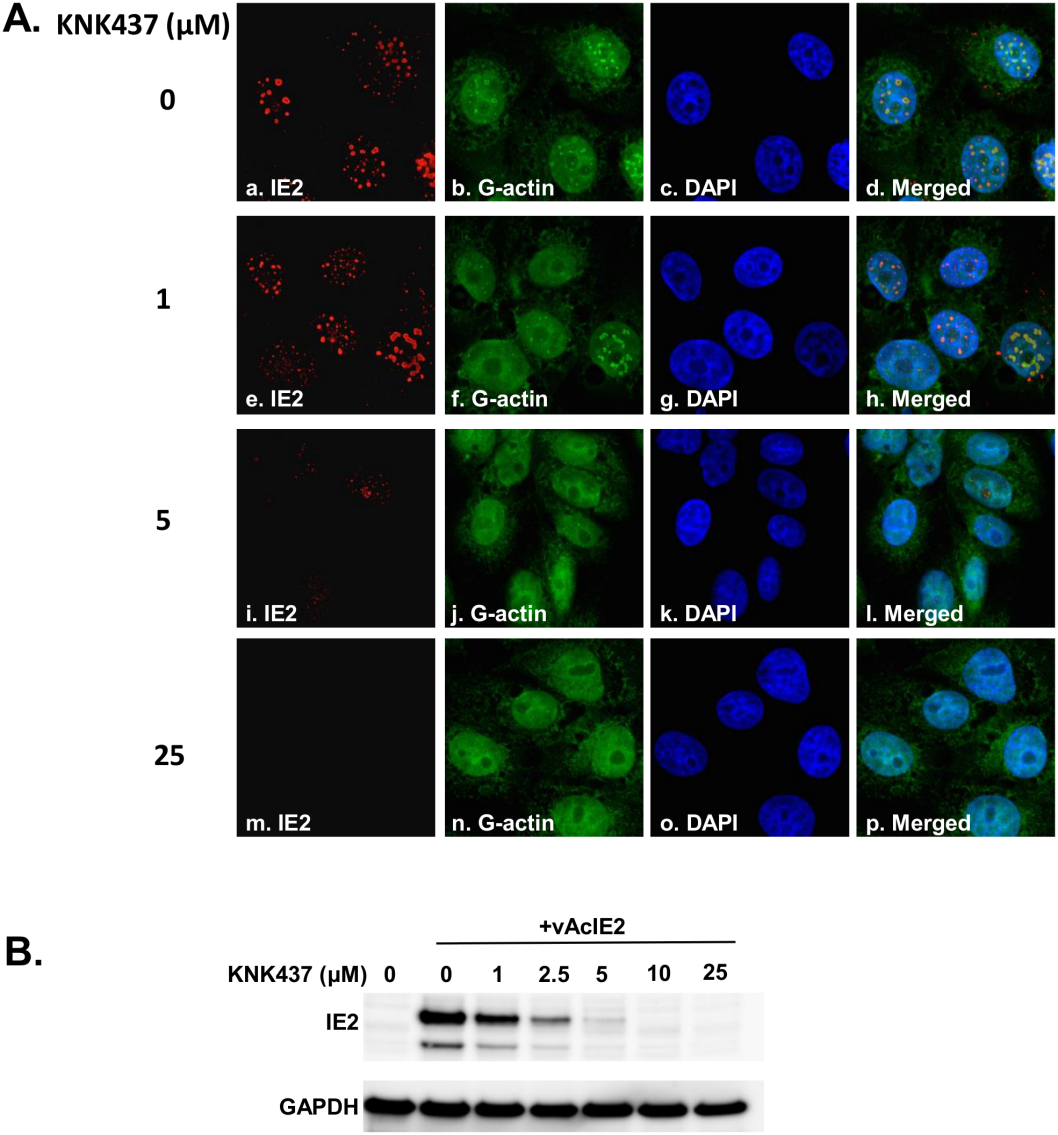
HSPs stabilize the IE2 protein. (A) Vero E6 cells were transduced with vAcIE2 virus together with KNK437 treatment at various concentrations. The transduced cells were fixed at 48 hpt for the detection of IE2 nuclear body formation. Immunofluorescence staining assays showed that, in the presence of KNK437 at concentrations up to 5 μM, smaller and fewer IE2 nuclear bodies were observed. Smaller IE2 nuclear bodies also failed to incorporate G-actin into the mature nuclear body structure. (B) vAcIE2-transduced cells were collected at 48 hpt for the detection of IE2 protein. Western blot analysis showed that the higher the concentration of KNK437, corresponding to greater inhibition of the heat shock response, the lower the levels of IE2 protein.

Given that HSPs play a central role in maintaining protein homeostasis by regulating the folding and degradation of client nascent peptides [42], we next investigated whether the interaction between HSPs and IE2 leads to the maintenance of IE2 protein stability, which contributes to *trans*-activation activity of IE2. Because we had previously determined that the formation of intact nuclear bodies was crucial for *trans*-activation activity [4] and that HSPs are usually important for maintenance of target protein conformations, we conducted immunofluorescence studies of the IE2 structure in the nucleus. Interestingly, treatment with 1 μM KNK437 was the lowest concentration at which we could still clearly observe the formation of regular IE2 nuclear bodies; at any higher concentration tested, the numbers and sizes of IE2 nuclear bodies declined significantly (Fig 5A), suggesting that the formation of functional nuclear bodies requires the proper stimulation of HSP expression in the transduced cells. To examine whether the disappearance of the IE2 nuclear body structures upon the elimination of HSPs was due to the degradation of IE2, a Western blot analysis was performed. As shown in Fig 5B, when HSP expression was blocked by KNK437, IE2 protein levels significantly decreased in a dose-dependent manner.

These studies suggest that without the proper stimulation of the heat shock response in the transduced cells, IE2 is completely degraded. Thus, these observations support the hypothesis that IE2 is a client protein for HSPs and that the interaction between HSPs and IE2 maintains the stability of IE2 protein, allowing IE2 to form a functional nuclear body structure that can strongly activate gene expression.

### IE2 levels are regulated by the ubiquitin-proteasome pathway

**Fig 6:**
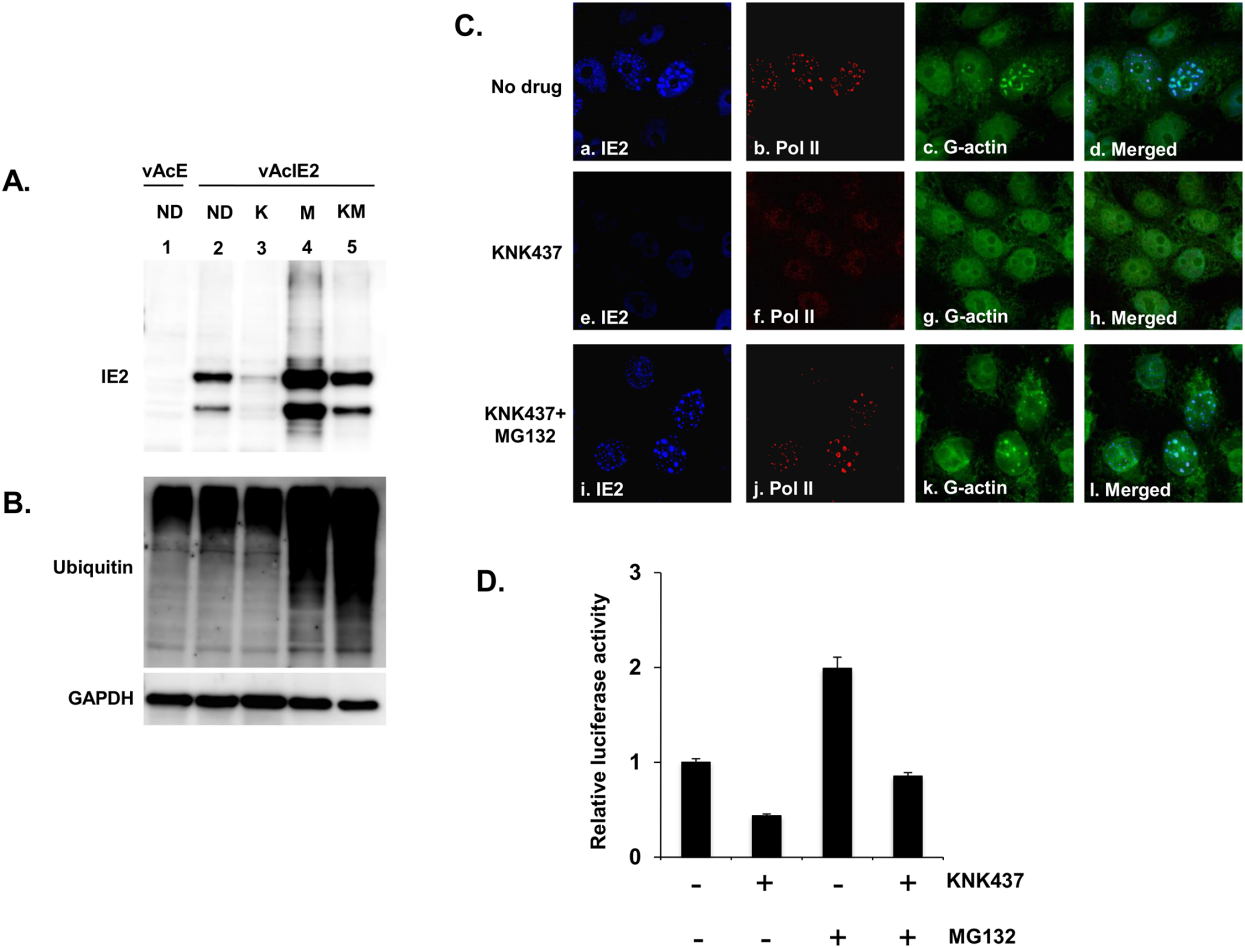
IE2 protein level is restored with the combination treatment of KNK437 and MG132. vAcIE2-transduced Vero E6 cells were treated with KNK437 (5 μM) and/or MG132 (2.5 μM) in various combinations. (A-B) Cell extracts were prepared at 48 hpt to detect the presence of IE2, ubiquitin and GAPDH proteins. Western blot analysis showed a significant recovery of IE2 protein levels in the presence of the proteasome inhibitor, MG132. ND: no drug, K: KNK437, M: MG132, KM: KNK437+MG132. (C) vAcIE2-transduced Vero E6 cells were fixed and examined by immunofluorescence staining. Panels *a* to *d* show that at 48 hpt, IE2 formed mature nuclear bodies that included a high concentration of G-actin (green) and enlarged, activated Pol II dots (red). KNK437 treatment alone (panels *e* to *h*) led to a failure in IE2 nuclear body formation as well as G-actin and activated Pol II recruitment, whereas for KNK437 in combination with MG132 (panels *i* to *l*), mature IE2 nuclear bodies could again be observed. (D) vAcIE2 and vAcL co-transduced Vero E6 cells were treated with drugs at the indicated combinations and collected at 48 hpt. The luciferase assay shows that IE2 *trans*-activation activity was also restored in the presence of MG132.

To investigate whether IE2 can be degraded by the ubiquitin-proteasome pathway following the down-regulation of the heat shock response, the proteasome inhibitor MG132 was used in combination with KNK437 to investigate the effects on IE2 protein levels. As shown earlier, treatment with KNK437 alone resulted in reduced IE2 levels (Fig 6A, lane 3). Upon combined treatment with KNK437 and MG132, we observed elevated levels of ubiquitin-conjugated proteins because of the reduced degradation functionality of the proteasome due to MG132 [43] (Fig 6B, lane 5), and compared with Fig 6A, lane 3, the IE2 protein level was also significantly restored (Fig 6A, lane 5). These observations further confirm that the induction of HSPs is essential for the stable presence of IE2 and that without the possible protection from HSPs, unstable IE2 may be actively removed by the proteasome.

Although the experiments in Fig 6A show that IE2 can be restored upon MG132 treatment, the formation of the IE2 nuclear body structure is more important for IE2 functionality [4]. To study the morphology of the IE2 nuclear body upon these treatments, immunofluorescence staining was performed. As shown in Fig 6C, without KNK437 treatment, IE2 nuclear bodies were observed with an accumulation of unique, granulated RNA polymerase II (Fig 6C, panel *a*-*d*) [4]. Conversely, no IE2 nuclear body structure was observed upon KNK437 treatment and, instead, normal, well-dispersed RNA polymerase II was observed (Fig 6C, panel *e*-*h*). Using the KNK437+MG132 combined treatment, IE2 protein levels were restored (Fig 6A) and, at the same time, a normal IE2 nuclear body structure, together with the presence of unique, granulated RNA polymerase II, was also observed (Fig 6C, panels *i*-*l*). Furthermore, because the IE2 structure can be recovered with dual drug treatment, the function of IE2 was also investigated. As shown in Fig 6D, without the drug treatment, IE2 *trans*-activated the *luciferase* gene expression driven by the CMV promoter (Fig 6D, column 1). This activity was suppressed by treatment with KNK437 (Fig 6D, column 2). Addition of MG132 enhanced gene activation (Fig 6D, column 3) and, interestingly, addition of both drugs rescued IE2 activity to a level very close to that without drug treatment (Fig 6D, compare column 1 and column 4). These studies further confirm that the heat shock response may be closely associated with the protection of IE2 from proteasome degradation.

### IE2 degradation is independent from its own ubiquitin E3 ligase activity

**Fig 7:**
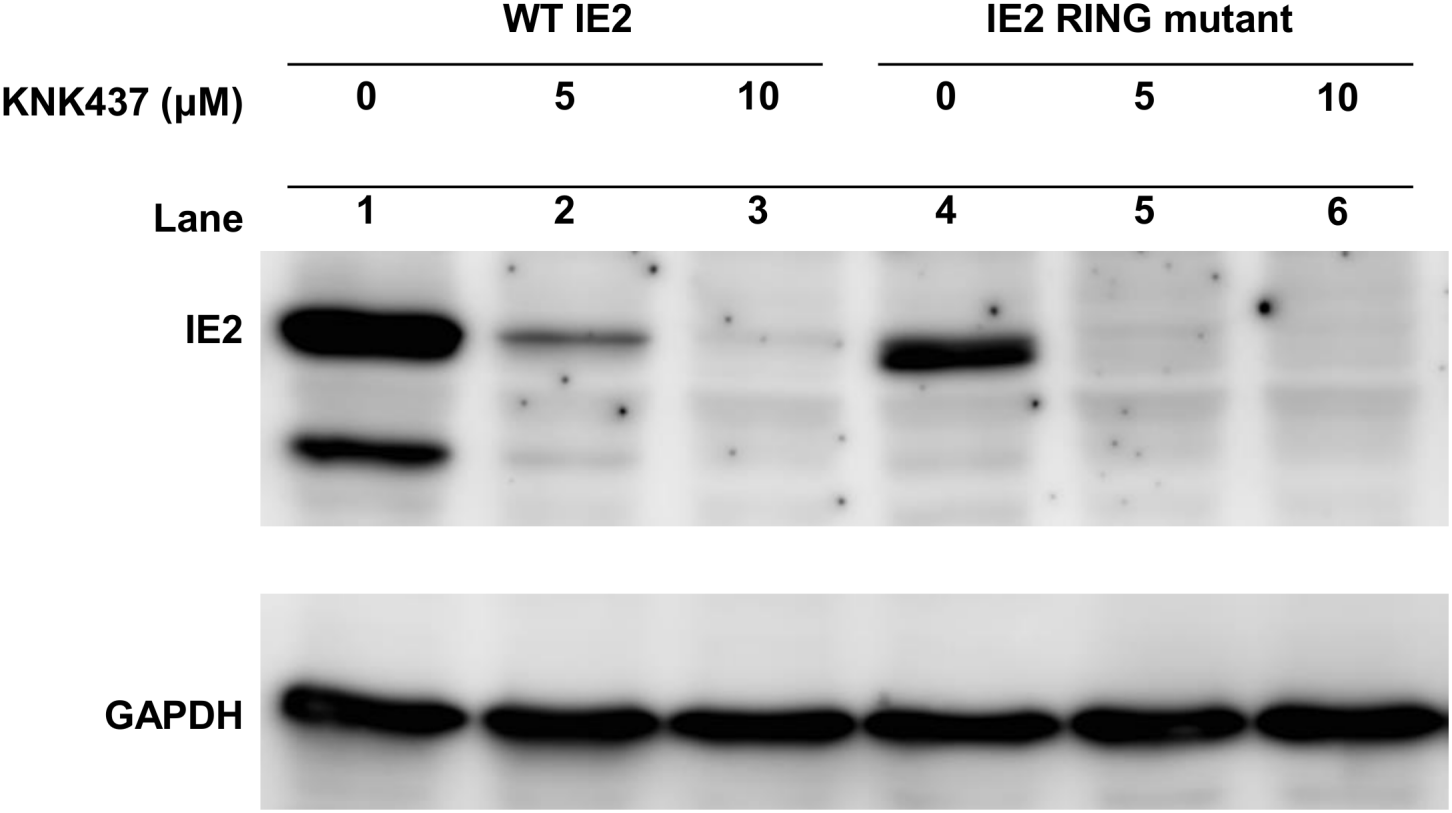
IE2 is degraded in the absence of its E3 ligase activity. Wild type (WT) IE2 and IE2C230S (containing a RING domain mutation)-expressing recombinant viruses were transduced into Vero E6 cells together with KNK437 treatment at various concentrations. The cell extracts were collected at 48 hpt for the detection of IE2 protein levels.

An auto-ubiquitylation and degradation mechanism was previously proposed when the IE2 RING domain mutant was found to be more sustained than wild-type IE2 in insect cells [14]. To determine whether degradation of IE2 is controlled by its own E3 ligase activity, we expressed both the wild-type IE2 and IE2 RING domain mutant, IE2C230S, and examined their protein levels in response to KNK437 treatment in Vero E6 cells. Similar to wild-type IE2, the protein levels of IE2C230S were also down-regulated in the presence of KNK437 (Fig 7), indicating that IE2 could still be degraded in the absence of its E3 ligase activity. The lower levels of IE2C230S protein (Fig 7, lane 4) compared to wild-type IE2 protein (Fig 7, lane 1) may result from the reduced *trans*-activation activity of the mutant protein at its own CMV promoter which drives IE2 [4]. Our results corroborate those of a study from 2011 [44], in which it was found that even though the IE2 RING domain mutant exists at higher levels, IE2 levels still gradually decreased after 4 hours post infection (hpi) during the virus infection cycle, suggesting that there is another regulatory pathway responsible for IE2 degradation.

### The interplay between HSC70 and IE2 during baculovirus infection

Although a previous study has shown that inhibition of the heat shock response decreased the levels of viral replication in infected cells [23], the viral factor(s) that are responsible for this activation and how they might interact with HSPs is still unclear in host insect cells. Based on our findings acquired from the study of mammalian cells, we further investigated whether similar interactions between HSPs and IE2 also occur in insect cells.

**Fig 8:**
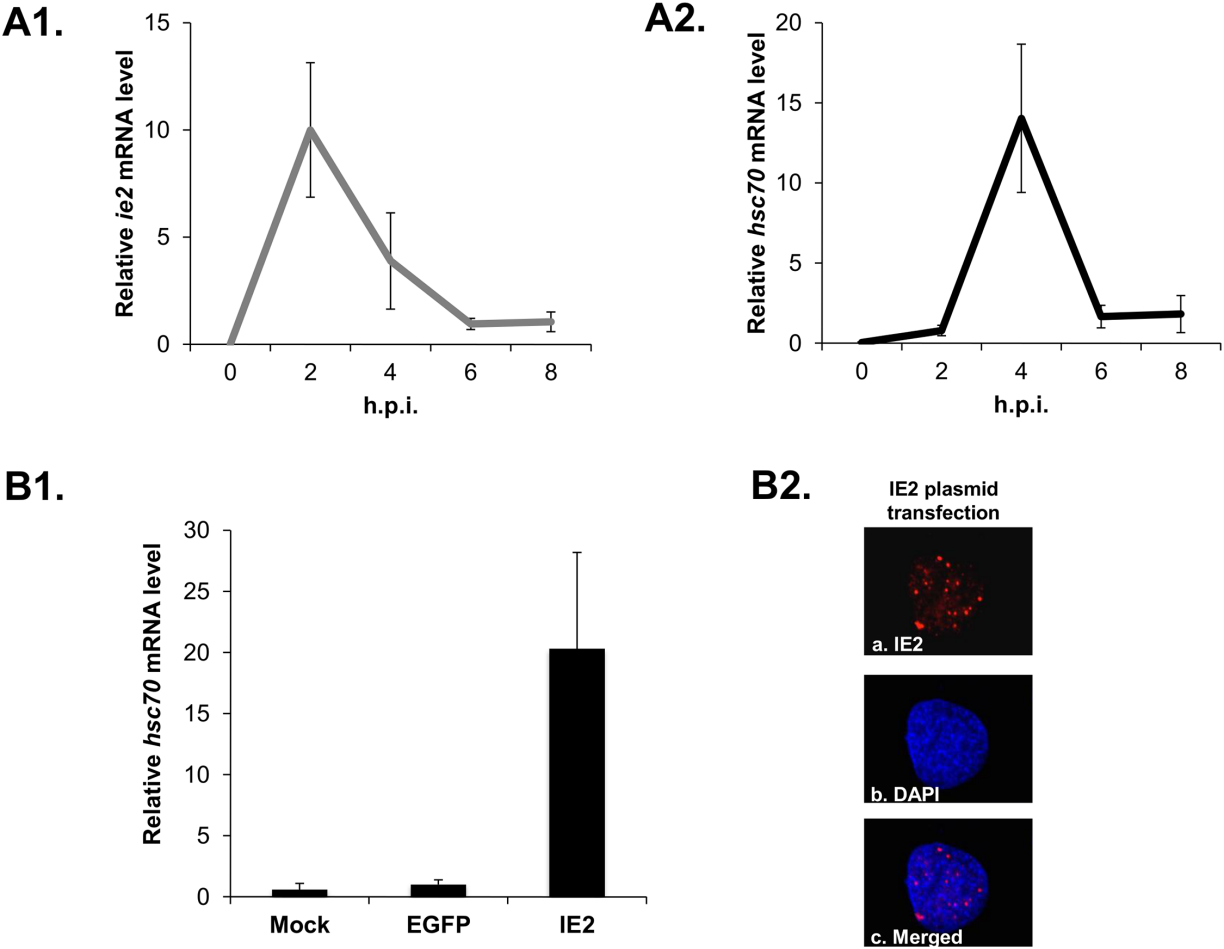
IE2 stimulates HSC70 expression during baculovirus infection in insect cells. (A) Total RNA was collected from vAcE-infected Sf21 cells at 0, 2, 4, 6, 8 hpi, and the expression patterns of *ie2* (A1) and *hsc70* (A2) genes were quantified by RT-qPCR using specific primers. (B) Plasmids expressing the *ie2* and *egfp* genes were transfected into Sf21 cells in the absence of virus. An up-regulated level of *hsc70* transcripts can be observed using RT-qPCR (B1). IE2 nuclear bodies can also be detected within the nucleus of the transfected cells (B2). In A1, A2 and B1, the relative *ie2* and *hsc70* levels were individually normalized to *actin* gene levels.

First, we examined the expression pattern of heat shock proteins and IE2 during baculovirus infection. Given that the heat shock cognate protein gene (*hsc70*) is the only gene that has been sequenced among the HSP/HSC70 family in *S*. *frugiperda* cells [24], and that it has been found to be transiently up-regulated during baculovirus infection [24, 25], we chose *hsc70* as a representative of the heat shock response in *S*. *frugiperda* cells. Transcripts from the infected cells were collected at 0, 2, 4, 6, 8 hpi and quantified by RT-qPCR. As shown in Fig 8A, expression of IE2 RNA rapidly increased after virus infection, reaching maximum levels at 2 hpi, followed by a gradual decline. In contrast, *hsc70* levels only began increasing at 2 hpi, reached maximum levels at 4 hpi, followed by a dramatic decrease. A lag in the expression of *hsc70* compared with that of *ie2* strengthens the possibility that IE2 is a stimulator for HSP induction.

To investigate this hypothesis further, we transfected an IE2-expressing plasmid into Sf cells in the absence of virus to exclude the interference of other viral factors. Our data show that the expression of IE2 resulted in a 20-fold stimulation of *hsc70* expression, whereas expression of a control foreign protein did not cause a similarly strong effect (Fig 8B). Together, our results indicate a stimulator role for IE2 on insect HSP induction. Given that IE2 is one of the very first viral genes expressed during the virus infection cycle, it is highly possible that IE2 is at least one of the major stimulators of the heat shock response upon viral infection. However, we do not exclude the possibility that other viral factors also contribute to this stimulation.

### HSPs stabilize the IE2 protein during baculovirus infection

**Fig 9:**
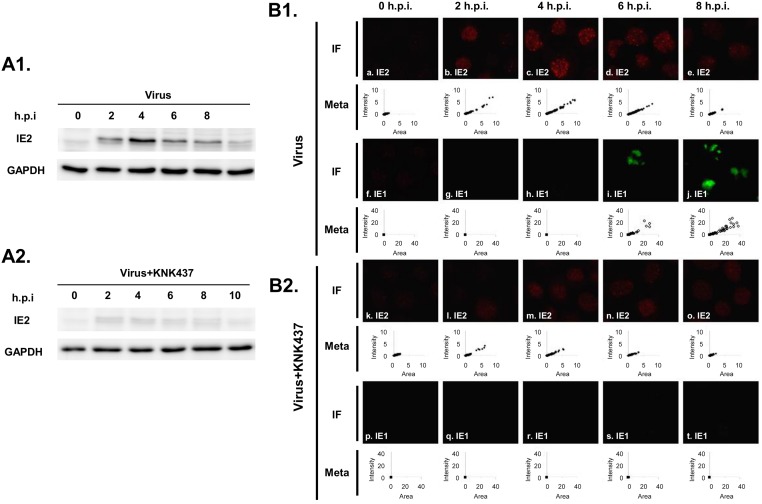
HSPs stabilize the IE2 protein during baculovirus infection. vABiIE1WF-infected Sf21 cells were treated with DMSO or KNK437, and the samples were collected at the indicated times. (A) Western blot analysis showing the expression pattern of IE2 and GAPDH without (A1) and with (A2) KNK437 treatment. KNK437 treatment led to a significantly decreased level of IE2 at various time-points. (B) Immunofluorescence staining assays followed by MetaMorph analysis show that both the intensity and formation efficiency of the IE2 and IE1 nuclear bodies decreased without (B1) and with (B2) KNK437 treatment.

Previously, baculovirus infection has been shown to result in a general shutdown of host gene expression, whereas *hsc70* is stimulated by an unknown mechanism [24]. Another independent study has also indicated, using a two-dimensional PAGE followed by mass spectrometry approach, that various HSPs are up-regulated in response to baculovirus infection [45]. Based on our findings regarding the IE2 and HSP relationship in a mammalian system, we hypothesized that HSPs stimulate IE2 protein stabilization. To verify this hypothesis, we examined IE2 protein levels upon treatment with a heat shock response inhibitor, KNK437, which has also previously been applied in an insect cell system [23]. As shown in Fig 9A, upon baculovirus infection of Sf cells, IE2 protein was detectable as early as 2 hpi, followed by a decrease after 4 hpi. In contrast, in the presence of KNK437, IE2 protein was mostly reduced at each time-point observed, indicating a supportive role of HSPs for IE2 during baculovirus infection.

The immunofluorescence staining data further confirmed this supportive role of HSPs, with IE2 starting to form multiple nuclear bodies at 2 hpi, and both the size and intensity of nuclear bodies gradually decreasing after 4 hpi (Fig 9B1, panels *a*-*e*). To monitor the virus life-cycle in response to this effect on IE2, we included another viral factor, IE1, as a representative indicator of virus progression. IE1 is known to be positively regulated by IE2 at the transcriptional level [46]. IE1 itself is also an essential transcriptional activator, inducing the production of several early viral proteins [47] through interactions with the origin of viral DNA [48], which is indispensable for virus amplification [49]. IE1 nuclear body began to increase at 6 hpi (Fig 9B1, panels *f*-*j*). This increase began much later than that of IE2 (Fig 9B1, panels *a*-*e*). With the addition of KNK437, the formation of IE2 (Fig 9B2, panels *k*-*o*) and IE1 (Fig 9B2, panels *p*-*t*) nuclear bodies significantly decreased, indicating that HSPs participated in regulating the formation of IE2 nuclear bodies during baculovirus infection, which in turn may affect the proper expression of other baculovirus genes. The intensities of the IE2 and IE1 nuclear bodies were also analyzed using MetaMorph image analysis software (Molecular Devices) for better comparability (Fig 9B, Meta).

### Stimulation of the heat shock response is crucial for baculovirus production

To demonstrate the importance of stimulating the heat shock response for the baculovirus infection cycle, we analyzed the expression of a late viral promoter as well as the production of budded virus at various time-points. Cells were either treated with KNK437 or DMSO, then infected with a recombinant virus expressing EGFP under a *p10* promoter. Expression of EGFP and the levels of budded virus were monitored at 12, 24, 48, 72 and 96 hpi.

**Fig 10:**
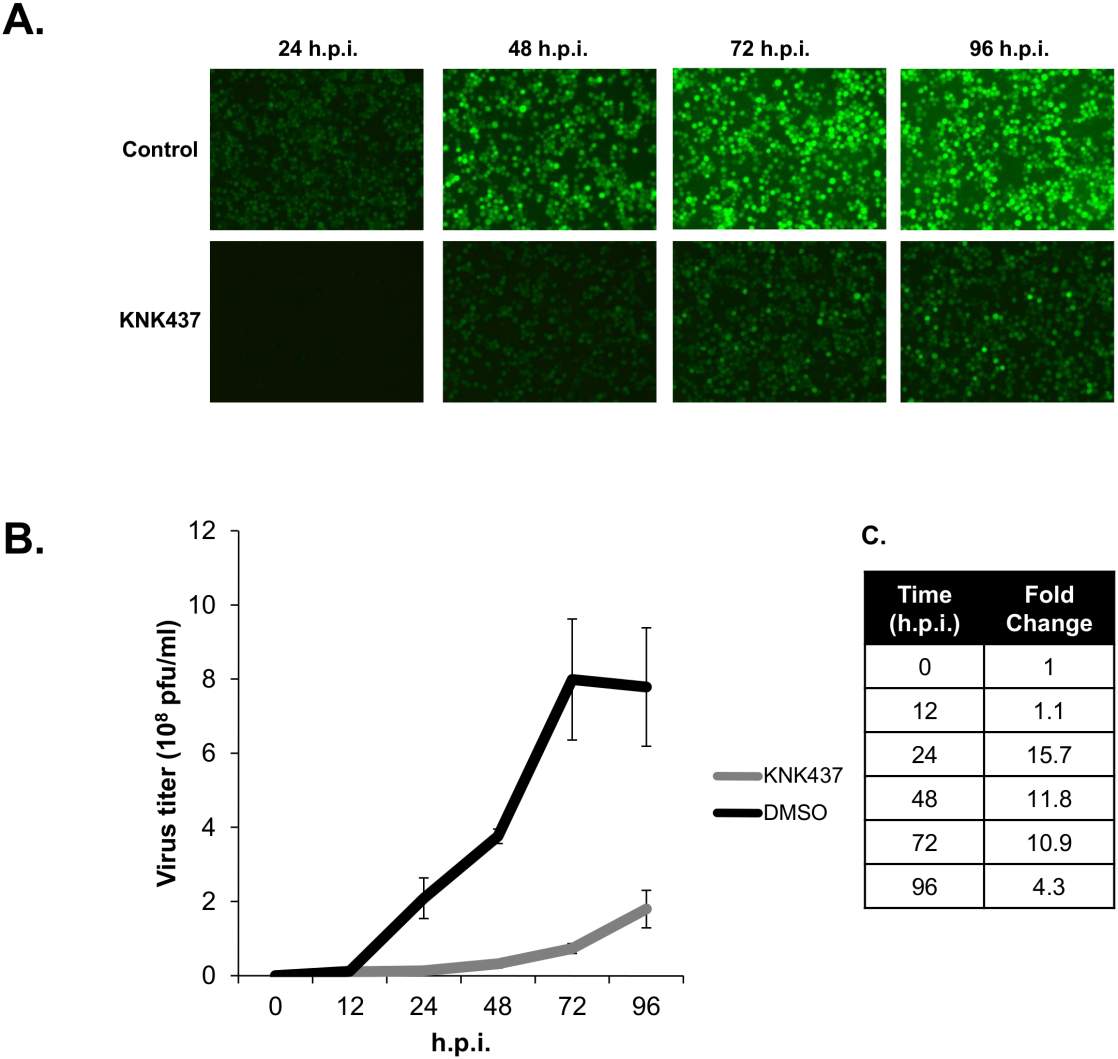
The heat shock response is important for baculovirus production. (A) Sf21 cells were treated with or without KNK437 and infected by vAcE recombinant AcMNPV expressing EGFP driven by the *p10* promoter. Green fluorescence images were taken at 24, 48, 72 and 96 hpi. (B) Budded viruses were collected from the supernatant of the infected Sf21 cells, and the virus titers were quantified by real-time quantitative PCR. KNK437 treatment resulted in a significantly lower level of virus production. When compared to the control group, a relative delay in virus amplification could also be observed. (C) n-Fold difference in virus titer between the KNK437-treatment group and the control group.

In the control infected cells, expression from the late viral promoter (as reflected by the EGFP signal in the infected cells) could be easily detected as early as 24 hpi and continued to rise steadily. In the KNK437 group, the increase in viral titer was delayed and progressed at a relatively slower rate, barely reaching the same expression level at 96 hpi as that of the control group at 24 hpi (Fig 10A).

To monitor the production of budded virus, the supernatants of the infected cells were collected and the amounts of virus were quantified using quantitative PCR. As shown in Fig 10B, the virus titer in the control group rose substantially after 12 hpi, reaching a plateau of 8x10^8^ pfu/ml at 72 hpi, whereas the virus titer increased at a much slower rate in the KNK437-treated group and reached a relatively low level of 1.8x10^8^ pfu/ml at 96 hpi. The differences between virus production are more significant at the earlier time-points; the titer was 15.7-fold lower in the presence of KNK437 at 24 hpi but only 4.3-fold lower at 96 hpi (Fig 10C). These results indicate the importance of the heat shock response for virus production, specifically during the early phase.

## Discussion

The heat shock response is stimulated in the early phase of baculovirus infection in insect cells [24, 25, 45, 50] and is crucial for the level of virus replication [45]. In this study, we demonstrate that baculovirus IE2 is an inducer of HSP expression in both insect and mammalian cells. HSPs may associate with the stabilization of IE2, and this interaction is essential for IE2 to form nuclear body structures as well as for gene *trans*-activation, both of which ensure proper progression of the viral infection cycle.

Baculovirus IE2-an immediate early gene product-is responsible for turning on early and late genes during virus infection in insect cells [15–17]. Our previous study also found that IE2 forms novel nuclear body structures where transcription-related factors, such as RNA Pol II and actin, are concentrated for the strong activation of promoters in mammalian cells [4]. In this study, by purifying the IE2 nuclear bodies, we identified heat shock proteins as IE2 interaction proteins, which are crucial for the proper functioning of IE2.

HSPs have a broad spectrum of cellular functions, such as protein quality control, protein complex assembly, protein activity regulation, intracellular protein trafficking, and cellular stress responses. Among all of these functions, the most notable is ensuring the proper folding and stability of client proteins. A long list of HSP client proteins can be found in the literature [51, 52]. In cancer cells, HSP70 and HSP90 have been found to be essential for WASF3 metastasis-promoting protein stability, which contributes to cancer cell migration and invasion [36]. HSP90 has also been found to directly bind to the ribosomal protein rpS3 to prevent it from degradation through a ubiquitin-proteasome pathway [53].

During virus infection, numerous host proteins have been found to be manipulated by viruses to profit their own gene expression and virus replication [27]. Exploiting the cellular stress response of the host cell is one of these strategies. HSPs are involved in various steps of virus infection, including the replication cycles, through interactions with viral proteins and by participating in capsid assembly and maturation [54, 55]. In influenza A virus, HSP70 negatively regulates the expression of viral proteins in infected cells by directly interacting with viral RNP, PB1 and PB2 subunits to block virus replication [56]. In herpes simplex virus 1, HSP90 is required for the proper nuclear localization of its viral DNA polymerase. HSC70/HSP70 has also been reported to form a virus-induced chaperone-enriched (VICE) domain that is induced by ICP22 [57] to remove stalled RNA Pol II and aberrant nuclear proteins for productive virus replication within the replication compartment [58–60].

Through our experiments, we found that IE2 induced strong HSP70 expression, and formed IE2/HSP70 complexes similar to the HSP70-enriched VICE domain. However, different from the VICE domains, although the stimulated HSPs were closely associated with IE2 stabilization probably through protecting it from degradation, this protection was not required if the ubiquitin-proteasome degradation was blocked. In this study, we found that addition of MG132 at the same time as virus transduction can assist IE2-mediated gene expression (Fig 6D, column 3). This suggests that MG132 may prevent IE2 from degradation, leaving more intact IE2 to activate gene expression from the CMV promoter. A similar result has also been found for another transcriptional activator, p53, for which upon MG132 treatment, the preserved p53 activator further up-regulated the expression of its targets p21/WAF1 [61]. These results suggest that there is a delicate balance between HSPs and proteasome degradation pathways, which regulates the presence and degradation of IE2 through time. This interaction is likely to also regulate the presence and degradation of other viral or host proteins, together coordinating overall virus-host interactions during the course of virus infection and ensuring that proper infection of the baculovirus can proceed in the host cells.

The heat shock response has long been known to be stimulated in the early phase of baculovirus infection in insect cells [24, 25, 45, 50]. Upon AcMNPV infection, the *hsc70* gene was first identified to be transiently up-regulated despite the fact that the majority of host cellular genes were down-regulated [24]. Such an early stimulation of the heat shock response has been suggested to facilitate baculovirus replication, although the mechanism regarding how the induction of HSPs contributes to virus production remains unknown [27, 45]. Since IE2 can still form a proper structure that is able to function under the combined treatment of KNK437 and MG132 (Fig 6), HSPs may not play a role in the proper folding of IE2. Instead, they may function to protect IE2 from degradation by the ubiquitin-proteasome pathway, allowing proper formation of the IE2 functional structure and *trans*-activation of gene expression. In addition, although IE2 is an E3-ligase, the IE2 RING domain mutant was still subjected to degradation upon heat shock response inhibitor treatment (Fig 7), suggesting that IE2 is not degraded by its own E3 ligase function.

Baculoviruses have been developed as an efficient tool for gene transfer in mammalian cells without obviously interfering with baculovirus gene expression [3]. In our studies, we first found that IE2 can stimulate HSP70 and HSP90 expression in Vero E6 cells. Upon inhibition of HSPs expression, IE2 protein was rapidly degraded by the proteasome. We have extended this research back to the original host, the *S*. *frugiperda* cell. Similar to what we found using the Vero E6 cells, transfection of the IE2-expressing plasmid can also stimulate endogenous *hsc70* gene expression. Since IE2 can activate gene expression in mammalian cells, these studies will result in better and broader applications of baculoviruses as a tool for other cell systems besides insect cells.

To date, little is known regarding which viral factors interact with HSPs or stimulate their expression during baculovirus infection [27, 62]. Considering the timing of IE2 and HSP expression in our studies, it is possible that IE2 is the stimulator of the heat shock response during baculovirus infection and that elevated HSPs maintain and stabilize the structure of its protein and nuclear bodies. This hypothesis is supported both by our data, which show that IE2 alone without the presence of virus is able to stimulate endogenous *hsc70* expression in insect cells (Fig 8), and by a similar result from another research group that has shown that IE2 can stimulate exogenous expression under the *Drosophila hsp70* promoter when the plasmids were introduced into the cells [63]. Up-regulated HSPs are obviously very important for viral DNA replication because treatment with a heat shock inhibitor resulted in the inhibition of viral DNA-binding protein (DBP) accumulation and down-regulation of viral DNA synthesis, which affected the production of viral progenies [45]. Furthermore, in our research, this treatment also resulted in down-regulation of IE1 nuclear body formation, which is commonly regarded as representative of virus progression. Although the down-regulation of IE1 nuclear body formation may be due to the combined effect of the lack of IE2 stimulation and the heat shock response, we cannot rule out the possibility that there is a direct role for HSPs in IE1 nuclear body formation.

In previous studies, the function of IE2 was found to be closely associated with viral DNA replication. A meticulous study using a transient DNA replication assay showed that although not strictly required, IE2 has a strong stimulating effect on AcMNPV origin-based replication [19]. Another independent study also demonstrated that IE2 nuclear body structures are the sites of viral DNA synthesis, as they co-localized with other replication factors such as DBP and LEF-3 [20]. These studies all point to the supporting role of IE2 in virus progression through regulation of several key viral factors. Combined with our results, these data further strengthen the importance of IE2 for facilitating virus amplification with the assistance of stimulated HSPs.

**Fig 11:**
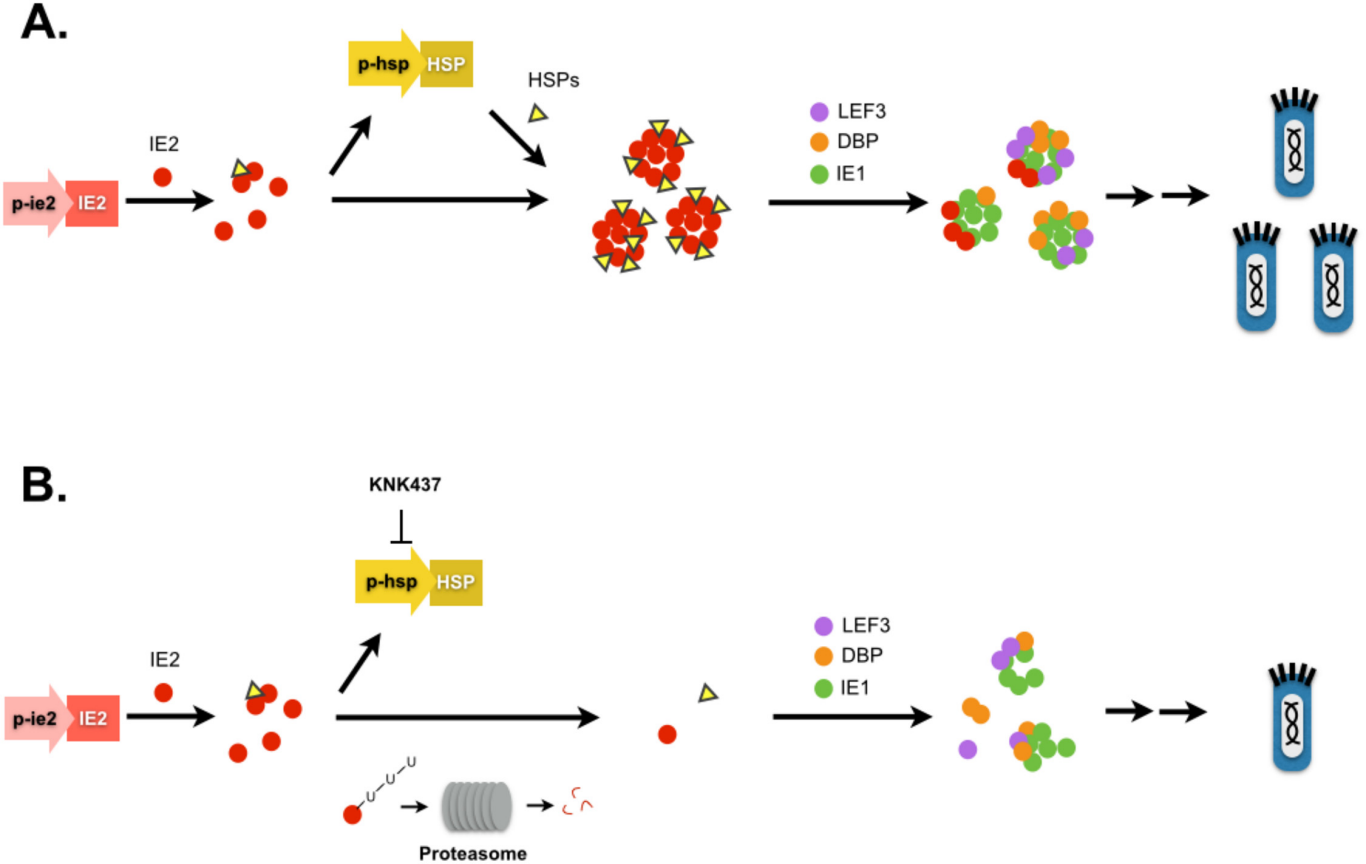
Schematic diagram of IE2 and heat shock protein interactions during baculovirus infection. (A) Upon virus infection, IE2 is expressed and stimulates heat shock protein expression. The elevated levels of heat shock proteins further sustain IE2 transcription center formation, most likely by protecting it from degradation. The IE2 transcription centers activate or recruit several additional viral factors including IE1, Lef3 and DBP [20], allowing the virus to maximize its amplification in the infected cells. (B) In the presence of KNK437-the heat shock response inhibitor-IE2 is rapidly degraded through the ubiquitin-proteasome pathway and fails to form mature transcription centers. The lack of IE2 nuclear body formation eventually contributes to lower levels of virus titer.

Here, we further establish the link between IE2 and HSPs during baculovirus infection and reinforce the importance of IE2 in the early phase of the baculovirus life-cycle (Fig 11). Application of the baculovirus expression system in mammalian cells relies on a strong and prolonged expression of target proteins using recognizable mammalian promoters, but limited expression levels have frequently been observed. With the use of baculovirus IE2, expression of a foreign gene can be significantly boosted in some mammalian systems to further advance the application of baculoviruses [4]. We have identified the first viral factor that induces HSP expression upon baculovirus infection. We show that HSPs and the ubiquitin-proteasome system work together to maintain the structure and function of IE2, and that HSPs may play an important role in protecting IE2 from proteasome degradation. However, although IE2 is an E3-ligase, it is not degraded by itself. Our experiments have demonstrated a mechanism revealing how HSPs assist virus infection at the early stage. Furthermore, our study also provides a unique example whereby the function of a viral factor can be identified and studied in one cellular system, such as a mammalian system, and then be verified in the original system, namely an insect system, to study its crucial role in the life-cycle of virus infection in the original host system.

## Supporting Information

S1 Table A list of primers used for gene amplifications or RNA quantifications. (DOCX)
