# Supplementary material for: Baculovirus IE2 Stimulates the Expression of Heat Shock Proteins in Insect and Mammalian Cells to Facilitate Its Proper Functioning
Source: PLoS One. 2016 Feb 10;11(2):e0148578. doi: 10.1371/journal.pone.0148578 (PMC4749218; doi:10.1371/journal.pone.0148578)
Supplement: S1 Table — (DOCX) [file pone.0148578.s001.docx]

**S1 Table: A list of primers used for gene amplifications or RNA quantifications**

| **Primer Name** | **Sequence (5’-3’)** |
| --- | --- |
| Luc-NcoI-F | GGCATTCCGGTACTGTTGG |
| Luc-SacI-R | CGATGAGCTCTTACACGGCGATCTTTCCG |
| IE2-F | ctaccggtcgccaccatgggtagtcgccaaatcaac |
| IE2-R | GATCCCATCGAATTCTTAGTGATGGTGATGGTGATGGTG |
| pKShE-F | gaattcgatgggatccaccgga |
| pKShE-R | GGTGGCGACCGGTAGCGC |
| pIE1-F | TATATAGTTGCTGATATCGGCCGACTGTTTTCG |
| IE1-F | AATTATCTCCATGATATTAAATTCGAATTTTTTATATTTACAATTTAGTTTTTG |
| pBacPAK-F | cggatccctgcaggcctcga |
| pBacPAK-R | ATCAGCAACTATATATTGATAGACATTT |
| L2-W-F | aaattcgaatttaattctgttcgaagtgctggcgtgagcaagggcgaggaga |
| W-FLAG-R | AATTATCTCCATGATTTACTTGTCATCGTCGTCCTTGTAGTCCTTGTACAGCTCGTCCATGC |
| pABiIE1-F | atcatggagataattaaaatgataaccatc |
| pABiIE1-R | ATTAAATTCGAATTTTTTATATTTACAATTTAG |
| HSP70-qF | CCGACAAGAAGAAGGTGCTG |
| HSP70-qR | TCTACCTCCTCAATGGTGGG |
| HSP90-qF | CAGATGCATTGGACAAAATCCG |
| HSP90-qR | ATGAACGCTTTGGTCCCAGAC |
| IE2-qF | AGCCGTCGCCGCATCAACTTTA |
| IE2-qR | CACGTAATACGTGACACCCTGACGA |
| ACTIN-qF | CACACCTTCTACAACGAGCTGC |
| ACTIN-qR | GAGTCCAGCACGATACCGGGT |
| 18S-qF | CATGGTGACCACGGGTGAC |
| 18S-qR | TTCCTTGGATGTGGTAGCCG |
| HSC70-qF | TGCCAAGCGTCTTATTGGTCGTG |
| HSC70-qR | ACGACAGCGTGGGTCACCTTCTT |
